# Impact of School-Based Physical Activity Intervention on Obesity and Physical Parameters in Children: A Systematic Review

**DOI:** 10.3390/children13010027

**Published:** 2025-12-23

**Authors:** Surendra Gupta, Purushottam Lal

**Affiliations:** 1 American Board of Pediatrics, Clinica Sierra Vista, Fresno, CA 93702, USA; guptas@clinicasierravista.org; 2 Pediatric Pulmonology, Yale New Haven Children Hospital, New Haven, CT 06511, USA

**Keywords:** physical activity, obesity, children, school-based programs

## Abstract

**Background:** Childhood obesity continues to pose a major global health challenge, and schools offer a structured and scalable setting for implementing physical activity programs. However, the effectiveness of these interventions remains inconsistent. This systematic review synthesizes evidence from school-based physical activity interventions and evaluates their impact on obesity-related parameters, physical activity levels, physical fitness, and cardiorespiratory fitness among children. **Methods:** A comprehensive search of PubMed, Scopus, and the Cochrane Library identified randomized controlled trials published between January 2015 and March 2025. Eligible studies included children aged 5–18 years and assessed school-based physical activity interventions. Outcomes included BMI, body fat percentage, physical activity levels (including MVPA), physical fitness, and cardiorespiratory fitness. Due to methodological heterogeneity, a narrative synthesis was conducted. **Results:** A total of 28 studies met inclusion criteria. Among the 16 studies reporting obesity-related outcomes, 7 demonstrated statistically significant improvements in BMI or BMI z-scores, while 6 of 16 (37.5%) showed no measurable effect. Reductions in body fat percentage were more consistently observed (5 of 6 studies). Both short-term (<6 months) and long-term (>12 months) interventions showed comparable proportions of studies with statistically significant BMI improvements (~50%). For physical activity outcomes, 5 of 11 studies reported increased MVPA, whereas others showed no significant change. Sedentary behavior outcomes were mixed, with only 2 of 6 studies demonstrating significant reductions. Improvements in physical fitness were reported in two-thirds of studies, while cardiorespiratory fitness improvements were inconsistent, with significant gains observed primarily in higher-intensity or well-structured programs. Across outcomes, several findings were statistically significant but modest in clinical magnitude. **Conclusions:** School-based physical activity interventions have the potential to improve select obesity-related parameters, particularly body fat percentage and BMI in a subset of studies. However, effects on MVPA, sedentary time, overall activity levels, and cardiorespiratory fitness remain variable. The effectiveness of these programs appears influenced by intervention structure, intensity, and adherence rather than duration alone. Future interventions should incorporate tailored, multi-component approaches to enhance both clinical relevance and long-term sustainability. While several effects were statistically significant, most were modest in magnitude. However, even modest improvements in BMI z-score, body fat percentage, and fitness can be meaningful at a population level, particularly when delivered through universal, scalable school platforms that reach large numbers of children.

## 1. Introduction

Physical activity during childhood is fundamental to healthy physical, cognitive, and psychosocial development, with benefits that extend well into adulthood. Global health agencies consistently emphasize its importance; for example, the World Health Organization (WHO) recommends that adolescents accumulate at least 60 min of moderate-to-vigorous physical activity (MVPA) daily to support optimal growth and reduce later cardiometabolic risk [1,2]. Physical activity also contributes to broader societal goals, including improved population health and sustainable development [3]. Despite these well-established benefits, physical activity levels among youth have declined over recent decades, largely driven by increasing screen time, digital engagement, and sedentary environments [4,5]. This decline is concerning, as inadequate physical activity contributes to poor cardiovascular risk profiles from early life [6] and negatively affects quality of life, self-esteem, and academic functioning [7,8,9].

Children and adolescents spend a large proportion of their daily time in school environments, making these settings uniquely positioned to influence physical activity behaviour. Their activity levels are shaped not only by structured PE but also by classroom routines, recess opportunities, and school infrastructure. The rise in childhood obesity further underscores the importance of daily movement. Obesity during childhood increases the likelihood of adult obesity four-fold and is associated with long-term metabolic, cardiovascular, and psychosocial complications [10,11,12,13,14,15]. Schools, where children spend most of their waking hours, offer a structured, equitable, and scalable setting for promoting physical activity. Traditional physical education (PE) curricula provide some activity exposure, yet research suggests that standard PE alone is often insufficient to counteract modern sedentary behaviors [16,17,18,19]. As a result, schools increasingly incorporate a range of movement-based strategies such as active classroom lessons, short activity breaks, active transportation initiatives, and playground modifications designed to integrate physical activity throughout the school day.

Although numerous reviews have examined school-based interventions, their findings remain inconsistent. Several reviews combined multi-component interventions that included nutrition education or behavioral counseling, making it difficult to isolate the independent effect of physical activity on obesity outcomes [20,21,22]. Others focused primarily on academic performance or fitness without thoroughly evaluating obesity-related parameters [21]. Furthermore, some meta-analyses report only modest or non-significant impacts on obesity or physical activity behaviors [23,24]. These inconsistencies highlight a lack of clarity regarding which school-based physical activity strategies are effective, under what conditions, and for which outcomes.

This reveals a clear research gap: there is limited consolidated evidence on school-based interventions that involve physical activity alone, excluding dietary or multi-component lifestyle elements. Existing studies also provide little comparison of how intervention characteristics—such as duration, type (e.g., structured PE, high-intensity sessions, activity breaks), and adherence—shape obesity, physical activity behavior, fitness, and cardiorespiratory outcomes. Understanding these isolated effects is essential for designing practical, scalable programs, especially in schools where resources may not support comprehensive lifestyle interventions.

In addition to these inconsistencies, contemporary frameworks such as the Active School Concept emphasize that school-based physical activity should be understood as a whole-school ecological model rather than isolated PE sessions. This model integrates physical education, active classroom environments, structured recess, active transport, teacher professional development, and supportive school policies to create a movement-rich culture across the full school day. Its theoretical foundation is aligned with Bailey et al.’s widely cited educational framework, which highlights the physical, cognitive, social, and affective domains through which school-based movement fosters holistic child development. According to Bailey (2006), effective school-based programs must consider not only activity volume and intensity but also pedagogical quality, inclusiveness, engagement, and teacher-led facilitation. However, despite the prominence of these frameworks, few randomized trials explicitly apply the Active School principles, and even fewer evaluate their isolated effects on obesity, physical activity levels, physical fitness, or cardiorespiratory fitness. This further reinforces the need to clarify how different physical activity-only strategies, delivered within the broader school environment, influence specific health-related outcomes in children [25,26].

Therefore, this systematic review aims to address this gap by synthesizing evidence from randomized controlled trials that implemented school-based physical activity interventions only and reported outcomes on obesity-related parameters, physical activity levels, physical fitness, and cardiorespiratory fitness. By isolating the impact of physical activity-specific strategies, this review provides a clearer understanding of what schools can realistically achieve through movement-focused programs and identifies which approaches demonstrate the most promising effects.

## 2. Methods

### 2.1. Study Design

The aim of this systematic review was to evaluate the impact of school-based physical activity interventions on a range of obesity-related outcomes and physical health parameters among children. The review was carried out in accordance with the PRISMA guidelines which are widely recognized for promoting methodological rigor and transparency in systematic reviews.

### 2.2. Eligibility Criteria

The studies included in this review were selected based on the following criteria:

Population: The target population consisted of children and adolescent aged 5 to 18 years who were enrolled in formal school settings.

Intervention: The interventions considered were school-based physical activity programs which included but were not limited to structured physical education sessions, classroom-based activity breaks, active transportation initiatives, and playground modifications.

Comparators: The comparator groups were participants following the usual curriculum of the school or a control group that received either no intervention or exposure to only minimal physical activity.

Outcomes: The primary outcomes assessed were obesity-related parameters such as body mass index (BMI), BMI z-score, body fat percentage, waist circumference, and skinfold thickness. The secondary outcomes included changes in physical activity levels, overall physical fitness, and cardiorespiratory fitness.

Study Design: only randomized controlled trials (RCT) were considered including both cluster RCT and single site RCT.

Language: the review included only those studies that were published in English.

Exclusion Criteria:

Studies were excluded if they

1. Included dietary, behavioral, or multi-component lifestyle interventions in combination with physical activity.
2. Were non-randomized, observational, cross-sectional, qualitative, or pilot studies without a control group.
3. Involved children with chronic disease-specific interventions (e.g., asthma-specific activity programs).
4. Were conducted outside formal school settings (e.g., community, after-school clubs, sports academies).
5. Did not report any obesity-related or physical activity-related outcomes.
6. Were not published in English.
7. Included participants younger than 5 or older than 18 years.

### 2.3. Information Sources and Search Strategy

For the PubMed search, we used a combination of Medical Subject Headings (MeSH) and free-text terms to ensure comprehensive retrieval of relevant studies. The search incorporated MeSH terms related to the school environment, including Schools, Students, Education, Primary, and Education, Secondary. Physical activity-related concepts were captured using terms such as Exercise, Motor Activity, Physical Fitness, Exercise Therapy, Sports, Play and Playthings, and Transportation to include interventions involving PE lessons, activity breaks, active play, and active commuting. Obesity and body composition outcomes were identified using the MeSH terms Obesity, Overweight, Body Composition, Body Fat Distribution, and Body Mass Index. To restrict the population to children and adolescents, we applied the MeSH terms Child, Adolescent, and Pediatrics. Study design filters were applied using the publication types Randomized Controlled Trial and Controlled Clinical Trial, as well as Clinical Trials as Topic to ensure methodological rigor. Alongside MeSH terms, we included relevant keywords such as “school-based,” “physical education,” “active lesson*,” “active break*,” “moderate-to-vigorous physical activity,” “MVPA,” “active transport*,” and “sedentary behavior” in the title and abstract fields to capture studies that may not be fully indexed under MeSH. This combined MeSH and keyword-based approach enabled a thorough and reproducible search of school-based physical activity interventions targeting obesity and activity-related outcomes in children.

### 2.4. Study Selection

**Figure 1:**
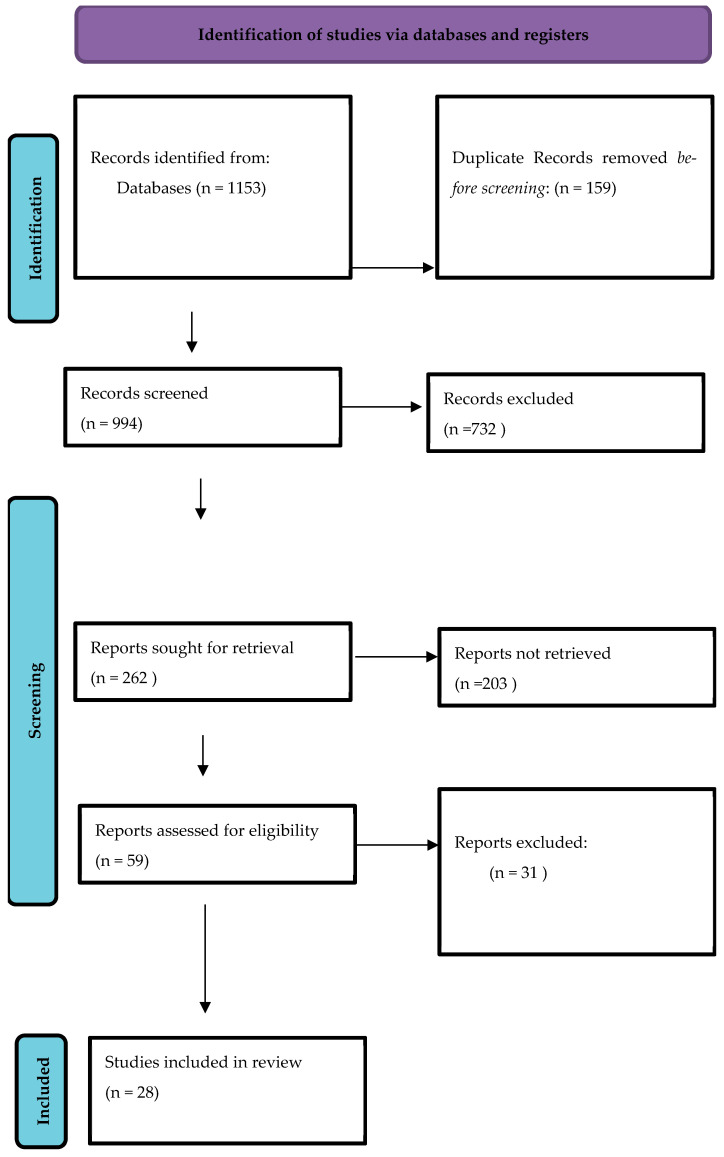
PRISMA flow diagram.

All identified studies were screened independently by two reviewers (SG and PL) in two phases: (1) title and abstract screening and (2) full-text review. Any initial disagreements were resolved through discussion. In rare instances where consensus could not be reached, the reviewer with greater methodological experience (SG) provided the final decision. A PRISMA flow diagram (Figure 1) outlines the study selection process.

### 2.5. Data Extraction

Data from included studies were extracted using a pre-designed standardized data extraction form. Extracted data included study characteristics (author, year, country); population details (age, sample size); type and duration of intervention, outcome measures and assessment tools and main findings related to obesity, physical activity, physical fitness, and cardiorespiratory fitness. Two reviewers independently extracted the data, and discrepancies were resolved through discussion.

### 2.6. Risk of Bias Assessment

The methodological quality of included studies was assessed using the Cochrane Risk of Bias Tool (RoB 1.0), evaluating sequence generation, allocation concealment, blinding, incomplete outcome data, and selective reporting. Risk of bias was rated as low, high, or unclear for each domain. Assessment was independently performed by two reviewers.

### 2.7. Data Synthesis

A narrative synthesis was undertaken due to substantial methodological heterogeneity across studies, including variability in intervention types, duration, outcome definitions, measurement tools, and reporting formats. These differences precluded the pooling of data for meta-analysis. Subgroup or sensitivity analyses were considered; however, the limited number of studies within specific intervention categories, together with inconsistent reporting of comparable outcome measures, prevented meaningful statistical subgrouping. The outcomes from the included studies were organized into four major domains each of which reflects a distinct aspect of the interventions impact:

Obesity parameters, which include indicators such as body mass index (BMI), body fat percentage, and waist circumference.

Physical activity levels, which are measured through variables like moderate-to-vigorous physical activity (MVPA), total activity counts, and sedentary time.

Physical fitness, which is assessed using metrics such as muscular strength, flexibility, and agility.

Cardiorespiratory fitness, which is evaluated through measures like VO_2_ max and PACER laps.

Where applicable, the duration of the intervention was further stratified into short-term (less than 6 months), medium-term (6 to 12 months) and long-term (more than 12 months) categories. This stratification allowed for a more nuanced comparison across subgroups, especially when examining the temporal effects of the intervention.

## 3. Results

A total of 28 randomized controlled trials met the inclusion criteria and were incorporated into the final synthesis [27,28,29,30,31,32,33,34,35,36,37,38,39,40,41,42,43,44,45,46,47,48,49,50,51,52,53,54]. Of these, 16 studies assessed at least one obesity-related parameter, 22 studies evaluated physical activity or sedentary behaviour, and 20 studies reported outcomes related to physical fitness or cardiorespiratory fitness. The majority of interventions were cluster RCTs (n = 25) conducted in school settings with entire classes or year groups randomized, while three were individually randomized trials [48,49,54]. Intervention duration varied widely, ranging from 12 weeks [42,49,52] to 36 months [35], with a median duration of 10 months. The characteristics and its outcomes of the included studies are summarized in Table 1 and Table 2 respectively.

### 3.1. Obesity

Across the 16 studies that reported obesity-related parameters, BMI or BMI z-score was the most common primary measure (14/16 studies). Among these, eight studies demonstrated statistically significant improvements following intervention—specifically Jarani et al. (2016) [31], Müller et al. (2019) [40], Pfeiffer et al. (2019) [41], Hollis et al. (2016) [30], Leahy et al. (2019) [39], Maglie et al. (2022) [48], Marsigliante et al. (2023) [54], and Meng et al. (2022) [49]. These studies largely incorporated structured, supervised, or higher-intensity activity components, such as specialized PE curricula [31,40], high-intensity interval training [37,47], or daily activity breaks integrated into classroom sessions [54].

By contrast, seven studies (50%) found no significant change in BMI, BMI z-score, or weight-related outcomes, including Lubans et al. (2016) [32], Tarp et al. (2016) [34], Donnelly et al. (2017) [35], Have et al. (2018) [37], Breheny et al. (2020) [46], and others. These interventions typically involved either lower-intensity activities, insufficiently structured PE modifications, or shorter exposure periods.

Body fat percentage, a more sensitive index of adiposity in growing children, was assessed in six studies [31,38,41,45,46,49]. Notably, five of these (83.3%) showed significant reductions—particularly in strength-training or skill-oriented PE interventions such as Ten Hoor et al. (2018) [38], movement-enhanced PE in Zhou et al. (2019) [45], and structured training programs in Meng et al. (2022) [49]. The only study that did not show change was The Daily Mile trial by Breheny et al. (2020) [46], which relied primarily on self-paced outdoor walking and may have lacked sufficient intensity to influence adiposity.

Waist circumference, assessed in six studies [32,34,35,48,49,54], demonstrated significant reductions in half (3/6), specifically, Maglie et al. [46], Marsigliante et al. [54], and Meng et al. [49], whereas studies such as Lubans et al. [32], Tarp et al. [34], and Donnelly et al. [35] reported no meaningful differences. Programmes with structured strength or high-intensity training were more likely to elicit central adiposity improvements.

The few studies evaluating skinfold thickness—including Müller et al. (2019) [40]—reported significant changes, reinforcing the value of body-composition-oriented outcomes over BMI alone.

When obesity outcomes were examined by duration, short-term (<6 months) programmes yielded significant improvements in 3/6 trials [31,39,49], medium-term (6–12 months) programmes in 5/8 trials [38,40,45,48,54], and long-term (>12 months) programmes in 1/2 trials [30,35]. These results suggest that programme content, structure, and intensity exert a stronger influence than duration alone, since effects in short-term and long-term programmes were comparable.

Overall, 6 of the 16 studies (37.5%) found no significant change in any obesity parameter [32,34,35,37,44,46], indicating substantial heterogeneity in the effectiveness of school-based physical activity programmes targeting adiposity.

### 3.2. Physical Activity

A total of 22 studies evaluated changes in physical activity behaviour after intervention. Moderate-to-vigorous physical activity (MVPA) was the most frequently assessed outcome (11 studies). Of these, five studies (45.5%) reported significant improvements—including Cohen et al. (2015) [27], Sutherland et al. (2016) [50], Lonsdale et al. (2017) [35], Belton et al. (2019) [53], and Seljebotn et al. (2019) [44]. These interventions typically featured structured PE sessions, professional development for teachers to optimize MVPA during class time, or movement-enhancing lesson designs. For instance, Lonsdale et al. [36] achieved notable increases in in-class MVPA by training teachers to incorporate active instruction strategies, although these gains did not translate into leisure-time MVPA.

Conversely, six studies (28,33,34,38,42,51) found no significant change in MVPA, including Action 3:30R [28], SCORES adaptations [33], and Girls on the Move [42]. These interventions frequently relied on extracurricular models, voluntary participation, or low-intensity activities—factors that likely limited behavioural impact.

Total physical activity counts (cpm) were reported in six studies [27,31,32,34,37,52]. Of these, only two—Cohen et al. (2015) [27] and Carlin et al. (2018) [52]—observed meaningful increases. Studies incorporating walking-based sessions or unstructured light-intensity activity often did not produce significant cpm changes.

Sedentary behaviour was assessed in six trials [28,36,38,44,51,52]. Significant reductions were observed in only two studies—Carlin et al. (2018) [52] and Lonsdale et al. (2017) [36]—while others showed no meaningful difference. Across studies, increasing MVPA did not consistently translate to reductions in sedentary time, underscoring the behavioural distinctness of these domains.

Programme duration contributed to variability in behavioural outcomes. Longer trials (>12 months), such as Sutherland et al. (2016) [50] and Belton et al. (2019) [53], were more likely to show sustained MVPA or total PA improvements, whereas shorter programmes (<20 weeks) rarely demonstrated significant gains [28,34,42]. Collectively, these findings highlight that behavioural change in activity patterns is complex and may require environmental restructuring, parental engagement, and behavioural-change strategies, in addition to school-based PA alone.

#### Physical Fitness and Cardiorespiratory Fitness

Fitness outcomes were reported in 20 studies, spanning muscular strength, power, endurance, agility, and cardiorespiratory fitness (CRF). Two-thirds of studies (66.7%) showed significant improvements in at least one fitness domain. PE-integrated strength programmes such as Ten Hoor et al. (2018) [38], high-intensity interval training programmes (Leahy et al., 2019 [39]; Meng et al., 2022 [49]), and multidimensional activity interventions (Zhou et al., 2019 [45]) consistently led to improved muscular power, endurance, agility, and composite fitness scores.

CRF outcomes, assessed in 11 studies using VO_2_max estimates, PACER laps, or shuttle-run performance, showed significant improvements in 5 studies—including Jarani et al. (2016) [31], Leahy et al. (2019) [39], Pfeiffer et al. (2019) [41], Zhou et al. (2019) [45], and Ketelhut et al. (2020) [47]. These interventions shared common features: structured aerobic elements, progressive intensity, and frequent supervised training sessions.

In contrast, interventions that relied solely on classroom movement integration [29,35], unstructured active breaks [37,46], or general promotion of PA without intensity progression [43] failed to improve CRF. This aligns with exercise physiology principles requiring adequate training loads, duration, and intensity to induce cardiovascular adaptations.

## 4. Risk of Bias

Risk-of-bias assessment (Table 3) revealed generally adequate random sequence generation, with most studies rated low-risk in this domain. However, due to the nature of school-based interventions, blinding of participants and personnel was uniformly high-risk in nearly all studies. Approximately one-third demonstrated high or unclear attrition risk for anthropometric or physical activity outcomes, often due to accelerometer non-compliance, missing fitness test data, or loss of participants during follow-up. Selective reporting risk was low in the majority of studies. Overall, the methodological quality was moderate, with study heterogeneity and incomplete adjustment for cluster effects representing the most frequent limitations.

## 5. Discussion

The findings of this systematic review indicate that school-based physical activity interventions yield modest but meaningful improvements in certain obesity-related parameters and physical fitness, whereas effects on MVPA and cardiorespiratory fitness remain inconsistent. These results must be interpreted within the context of substantial methodological heterogeneity across included trials, which appears to influence both the magnitude and reliability of their reported effects. Differences in intervention structure, delivery mode, and fidelity were considerable across studies [27,28,29,30,31,32,33,34,35,36,37,38,39,40,41,42,43,44,45,46,47,48,49,50,51,52,53,54], and these factors likely contributed more to outcome variability than intervention duration alone—an aspect often emphasized in earlier work [11,20,21,22,23,24]. Programs incorporating structured, supervised, and intensity-defined sessions demonstrated clearer benefits for body fat percentage and physical fitness, whereas interventions that depended on sporadic activity breaks or low-intensity classroom movement showed weaker or null outcomes. This aligns with established physical activity guidelines emphasizing that health benefits in children depend not only on accumulating activity minutes but on appropriate intensity and quality of movement [1,2]. The term ‘modest’ reflects effect sizes that, while small at the individual level, are epidemiologically meaningful when achieved across large school populations. Even small shifts in BMI z-score or reductions in percent body fat during childhood have been associated with lower cardiometabolic risk later in life. Therefore, these modest improvements may still hold public health relevance, particularly when interventions are low-cost and broadly implementable.

A major source of heterogeneity derives from the broad range of measurement tools used for physical activity and adiposity outcomes. Accelerometry protocols differed widely in epoch length, wear-time criteria, cut-points, and data-processing decisions, resulting in limited comparability across studies [27,28,29,30,31,32,33,34,35,36,37,38,39,40,41,42,43,44,45,46,47,48,49,50,51,52,53,54]. Studies using self-report instruments likely introduced recall and social desirability bias, particularly among older children and adolescents [9,10]. Similarly, obesity-related outcomes were captured using diverse methods—including BMI, BMI z-scores, waist circumference, skinfolds, and body fat percentage—each varying in sensitivity to change during growth. Trials incorporating more sensitive composition metrics such as skinfolds and body fat percentage were more likely to detect significant improvements [31,38,41,45,49], whereas studies relying solely on BMI often reported null findings. Moreover, because most trials used cluster designs involving schools or classrooms, appropriate intraclass correlation (ICC) adjustments were essential; however, many studies did not report ICCs or adjust for clustering, increasing the risk of type I error and inflating apparent intervention effects [29,30,31,32,33,34,35,37,38,39,40,41,42,43].

Adherence, fidelity, and contamination were additional methodological concerns. Several interventions required teachers to deliver added activity sessions, and adherence varied widely, often without adequate reporting [27,28,29,30,31,32,33,34,35,36,37,38,39,40,41,42,43,44,45,46,47,48,49,50,51,52,53,54]. Contamination of control groups—wherein schools adopt competing health-promotion activities—was likely in several cluster trials and may have attenuated reported intervention effects. These issues highlight the need for future RCTs to incorporate systematic fidelity monitoring, transparent reporting of compliance, and analytic strategies that account for school-level influences and contamination risks [20,21].

Compared with previous systematic reviews that frequently evaluated multi-component interventions incorporating physical activity alongside dietary modification, behavioural counselling, or whole-school health promotion strategies [20,21,22,23,24], the present review isolates the effects of physical activity-only school-based programs. This narrower focus provides a novel contribution to the existing literature by identifying which health outcomes are achievable through movement alone, without the influence of nutritional or behavioural components that typically amplify effects. By using explicit denominators, clear categorization of study outcomes, and updated evidence through 2025, this review offers greater transparency and interpretability than earlier syntheses, clarifying what schools can realistically expect from movement-only interventions.

These findings can also be contextualized within the Active School Concept, which proposes that school-based activity should extend beyond scheduled PE sessions and encompass a whole-school movement culture. When interpreted through this lens, our results suggest that many interventions included in this review did not fully operationalize Active School principles. Bailey et al.’s educational framework similarly argues that high-quality physical activity opportunities require structured, engaging, and pedagogically sound experiences that target multiple domains of development. Several of the trials showing stronger effects—particularly those incorporating structured aerobic sessions, high-intensity intervals, or skill-based PE—align more closely with these frameworks, emphasizing intentional design and teacher-led delivery. Conversely, interventions relying solely on low-intensity movement breaks or unstructured activities appear insufficient to activate the full spectrum of mechanisms proposed by the Active School and Bailey frameworks. This suggests that intervention success may depend not only on duration or frequency but on the integration of multi-domain, whole-school design principles that enhance both movement quality and contextual support [25,26].

The theoretical contextualization of findings also warrants careful interpretation. The inconsistent impact on MVPA and sedentary behaviour aligns with the ActivityStat hypothesis, which posits that individuals compensate for increases in activity during certain parts of the day by reducing activity elsewhere [55]. Several trials reported increases in MVPA during structured PE lessons but no corresponding improvement in daily MVPA [31,34,40], suggesting potential compensatory declines during recess or after school. Similarly, parents may reduce opportunities for out-of-school activity when they perceive their children as sufficiently active at school, which aligns with observed compensatory patterns [56,57]. While the present review does not directly test the ActivityStat hypothesis, the observed trends warrant future experimental research integrating both behavioural and physiological monitoring to determine the extent of movement compensation in school-aged populations. Although high-intensity activity is most effective for improving fitness and adiposity, combining it with enjoyable, socially supportive PE experiences may enhance long-term adherence. Schools can achieve this hybrid model by integrating short HIIT bouts within enjoyable skills-based PE lessons.

The heterogeneity in cardiorespiratory fitness outcomes reflects variation in training specificity, intervention intensity, and supervision. Studies including structured aerobic exercise or high-intensity interval training demonstrated clearer improvements in VO_2_ max and aerobic fitness z-scores [39,47,49], whereas programs relying on general physical activity without explicit aerobic components showed minimal change [29,34,35,37,40,43]. These findings reinforce long-standing exercise physiology principles, wherein aerobic adaptations require appropriately targeted intensity and progression rather than simple increases in movement volume [5,58,59,60,61,62].

This review has several limitations. First, the significant heterogeneity in intervention definitions, activity characteristics, measurement tools, and analytic strategies precluded the conduct of a meta-analysis. Instead, a vote-counting approach was used, which allows identification of direction of effect but cannot estimate effect magnitude, precision, or account for variation in study quality [20,23]. Vote-counting may therefore underestimate small but consistent intervention effects. Second, the broad category of “physical activity intervention” remains heterogeneous across studies, ranging from PE curriculum changes to activity breaks, playground redesign, and walking programs, limiting comparability. Third, adherence and fidelity were inconsistently reported, restricting interpretation of null results. Fourth, most studies used cluster RCT designs but did not consistently adjust analyses for clustering, increasing the risk of biased effect estimates. Fifth, publication bias may favour positive findings, as grey literature and non-English language studies were excluded. Despite these limitations, this review provides the most focused synthesis to date on the isolated impact of school-based physical activity interventions, clarifying the outcomes most responsive to movement-only programming. Although necessary due to methodological heterogeneity, the vote-counting approach has inherent limitations. It does not incorporate effect sizes or variance and may underestimate small but consistent intervention effects that a meta-analysis could detect if comparable quantitative data were available [63,64,65].

Overall, the evidence indicates that school-based physical activity interventions can improve body composition—particularly body fat percentage—and aspects of physical fitness, while their influence on MVPA and cardiorespiratory fitness remains mixed. Future research should prioritize harmonized measurement protocols, rigorous fidelity monitoring, full adjustment for cluster designs, and integration of family or community components to reduce compensatory behaviours. Additionally, interventions explicitly incorporating structured aerobic intensity or progressive overload may better optimize both fitness and MVPA outcomes [66,67,68,69,70,71]. Such refinements can strengthen the evidence base and guide the development of more effective school-based physical activity strategies worldwide. Across outcomes, intervention structure and intensity consistently emerged as stronger determinants of success than programme duration, underscoring the importance of high-quality, well-designed activity delivery.

## 6. Conclusions

School-based physical activity interventions demonstrate modest and selective benefits, with the most consistent improvements seen in body fat percentage and certain fitness measures, while effects on BMI, MVPA, sedentary behaviour, and cardiorespiratory fitness remain mixed. These findings suggest that physical activity alone has limited impact on broad health outcomes without adequate structure, intensity, and implementation fidelity. Future research should prioritize standardized physical activity measurement protocols, consistent fidelity assessments, and rigorous adjustment for cluster effects, while also exploring dose–response relationships and compensatory behaviours to identify the specific intervention characteristics that produce meaningful and sustained health improvements in children.

## Figures and Tables

**Table 1:** Characteristic of studies included for systematic analysis.

Author (Year)	Study Design	Sample Size (n)	Age (Years)	Duration	Intervention Focus
Cohen (2015)	Cluster RCT	460	8–9	12 months	PA, CRF, anthropometry
Jago (2015)	Cluster RCT	571	11–12	20 weeks	Dance sessions
De Greeff (2016)	Cluster RCT	499	8	24 months	Active academic lessons
Hollis (2016)	Cluster RCT	985	11	24 months	School-based PA programme
Jarani (2016)	Cluster RCT	760	7–10	5 months	Exercise- vs. game-based PE
Lubans (2016)	Cluster RCT	361	12–14	20 weeks	ATLAS programme
Sutherland (2016)	Cluster RCT	985	14	24 months	PA during school day
Tarp (2016)	Cluster RCT	632	12–14	20 weeks	PA periods, recess, homework
Donnelly (2017)	Cluster RCT	1902	8	3 years	Active academic lessons
Lonsdale (2017)	Cluster RCT	1421	12–13	7–8 months	AMPED teacher-led PA
Sutherland (2017)	Cluster RCT	111	5–7	6 months	Modified SCORES
Carlin (2018)	Cluster RCT	197	11–13	12 weeks	Peer-led brisk walking
Have (2018)	Cluster RCT	450	7	12 months	PA integrated into math lessons
Ten Hoor (2018)	Cluster RCT	508	11–15	12 months	Strength training + motivation
Belton (2019)	Cluster RCT	534	12–13	24 months	Y-PATH
Jago (2019)	Cluster RCT	252	8–10	15 weeks	Action 3:30R
Leahy (2019)	Cluster RCT	68	16	14 weeks	Teacher-led HIIT
Müller (2019)	Cluster RCT	746	9–14	10 months	Multidimensional PA
Pfeiffer (2019)	Cluster RCT	1519	12	17 weeks	Girls on the Move
Robbins (2019)	Cluster RCT	1519	10–15	17 weeks	Girls on the Move (PA focus)
Seibert (2019)	Cluster RCT	4894	11	9 months	CDC PA strategies
Seljebotn (2019)	Cluster RCT	447	9–10	10 months	Active lessons + recess + homework
Zhou (2019)	Cluster RCT	680	12–13	8 months	CHAMPS intervention
Breheny (2020)	Cluster RCT	2280	7–10	12 months	Daily Mile programme
Ketelhut (2020)	Cluster RCT	48	11	3 months	HIIT during PE
Maglie (2022)	RCT	160	10–11	6 months	PE + sports enhancement
Marsigliante (2023)	RCT	310	8–10	6 months	Daily 10 min active breaks
Meng (2022)	RCT	36	10–13	12 weeks	HIIT vs. moderate training

PA = physical activity; CRF = cardiorespiratory fitness; PE = physical education; HIIT = high-intensity interval training.

**Table 2:** Study outcomes for obesity and physical activity-related parameters.

First Author (Year)	Intervention	Outcomes Measured	Result	Summary
Cohen (2015) [25]	Supporting Children’s Outcomes using Rewards, Exercise and Skills (SCORES)	Total physical activity (cpm)	54.2 (−10.3, 118.6)	Significantly higher improvement in MVPA and cardiorespiratory fitness in the intervention group compared to control group.
		MVPA (mins/day)	**12.7 (5.0, 20.5)**	
		20 m multistage fitness test (laps)	**5.4 (2.3, 8.6)**	
Jago (2015)	Bristol Girls Dance Project	Weekday MVPA	−1.52 [−4.76 to 1.73]	No significant difference in physical activity was observed between the two groups.
		Mean weekend day minutes of MVPA	−1.75 [−7.51 to 4.01]	
		Mean weekday (CPM)	−2.44 [−25.25 to 20.38]	
		Mean weekend (CPM)	−4.11 [−61.07 to 52.86]	
		Proportion of girls meeting 60 min MVPA per weekday	−1.18 [−1.82 to 0.76]	
		Proportion of girls meeting 60 min MVPA per weekend day	−1.11 [−2.39 to −0.52]	
		Mean weekday sedentary (mins)	−6.79 [−23.60 to 10.03]	
		Mean weekend sedentary (mins)	0.62 [−22.42 to 23.66]	
De Greeff (2016)	MVPA		Post-intervention values	No significant difference in cardiorespiratory and muscular fitness was observed between the control and intervention group.
		10 5 m shuttle run (s)	23.3 (2.3)	
		20 m shuttle run (stage)	4.7 (2.0)	
		Standing broad jump (cm)	129.7 (21.4)	
		Sit-ups (n)	16.7 (4.9)	
		Handgrip strength (kg)	**15.8 (3.7)**	
Hollis (2016)	Physical Activity 4 Everyone		Changes in adiposity outcomes; mean (95% CI)	A statistically significant improvement in adiposity outcomes was seen in children complying to the physical activity program after 2 years.
		Weight (kg)	**61.08 (59.83, 62.34)**	
		BMI (kg.m^−2^)	**21.86 (21.34, 22.37)**	
		BMI z-score	**0.69 (0.54, 0.84)**	
Jarani (2016)	Exercise based physical education sessions Game-based physical education sessions		Intervention effect in exercise-based group (95% CI)	Significant improvements in physical fitness were observed after the integration of exercise-based physical education sessions in elementary school children.
		VO2max (ml)	**2.0 (1.5; 2.4)**	
		10 × 5 m shuttle run (s)	**−0.9 (−1.3; −0.5)**	
		Standing long jump (cm)	**4.2 (2.0; 6.4)**	
		Sit-and-reach (cm)	−0.1 (−0.6; 0.4)	
		BMI (kg.m^−2^)	**−0.3 (−0.5; −0.1)**	
		Body fat (%)	**−0.4 (−0.6; −0.2)**	
		Physical activity (score)	0.1 (0.02; 0.2)	
Lubans (2016)	Active Teen Leaders Avoiding Screen-time’ (ATLAS)		Adjusted difference in change, Mean (95% CI) from baseline to 18 weeks	After an 18-month follow-up period, no significant intervention-related changes were observed in BMI, waist circumference muscle strength, or physical activity. However, the intervention significantly improved competency in resistance training skills.
		BMI (kg.m^−2^)	0.07 (−0.34, 0.38)	
		BMI-z scores	**0.04 (−0.07, 0.14)**	
		Waist circumference (cm)	0.3 (−0.7, 1.4)	
		Physical activity	0.1 (−0.8, 1.0)	
		Grip strength (kg)	0.3 (−0.7, 1.2)	
		Push-ups (repetitions)	0.5 (−0.6, 1.6)	
		Resistance training skill competency	**5.9 (4.5, 7.3)**	
Sutherland (2016)	Physical Activity 4 Everyone		Difference between control and intervention (95% CI)	The intervention successfully enhanced students’ engagement in physical activity.
		MVPA	**7.0 (2.68, 11.4)**	
		Moderate physical activity	**4.5 (2.0, 7.0)**	
		Vigorous physical activity	**2.5 (0.3, 4.8)**	
Tarp (2016) [32]	Physical activity periods, recess, and homework, and active transportation	Cardiorespiratory fitness (distance, m)	9.4 (−3.7, 22.4)	The intervention had no significant impact on cardiorespiratory fitness, physical activity, and anthropometric measures.
		Waist circumference (cm)	0.7 (−0., 2.1)	
		BMI	−0.1 (−0.2, 0.0)	
		Physical activity level (cpm)	5 (−30, 41)	
		Overall MVPA (minutes/day)	1.2 (−3.9, 6.3)	
Donnelly (2017)	Academic Achievement and Physical Activity Across the Curriculum		*p*-value between difference in both groups post intervention	No significant difference in body composition and cardiorespiratory fitness was observed between the control and intervention group.
		BMI percentile	0.08	
		Waist circumference	0.32	
		PACER laps	0.27	
Lonsdale (2017)	Activity and Motivation in Physical Education (AMPED)		Intervention-control adjusted difference in change (95% CI)	The intervention successfully enhanced students’ engagement in MVPA during lessons, demonstrating its effectiveness in promoting physical activity within structured school settings. However, its influence on leisure-time physical activity was minimal.
		PE lessons MVPA Sedentary Light Moderate physical activity Vigorous physical activity	Physical education lessons **5.66 (4.71 to 6.63)** **−11.11 (−12.63 to −9.59)** **5.36 (4.46 to 6.24)** **2.54 (2.07 to 3.01)** **3.09 (2.48 to 3.71)**	
		Leisure time MVPA Sedentary Light physical activity Moderate physical activity Vigorous physical activity	Leisure time **−1.09 (−1.87 to −0.31)** 0.92 (−0.28 to 2.13) 0.17 (−0.47 to 0.81) **−0.70 (−1.17 to −0.22)** −0.39 (−0.79 to 0.01)	
Sutherland (2017)	Modified Supporting Children’s Outcomes using Rewards, Exercise and Skills (SCORES)		Adjusted difference between treatment group (95% CI)	No significant difference in total MVPA and moderate physical activity was observed between the two groups. However, a statistically significant increase was observed in vigorous physical activity in the intervention group.
		Total MVPA	1.96 (–3.49, 7.41)	
		Vigorous activity	**2.19 (0.06, 4.32)**	
		Moderate activity	–0.23 (–3.84, 3.37)	
Carlin (2018)	Peer-led brisk walking intervention	Time (min/day)	*p*-value between difference in both groups post intervention	Increased walking during the intervention led to significant improvement in time spent in physical activity and reduced sedentary time.
		Sedentary	**0.013**	
		Light physical activity	**0.018**	
		Moderate physical activity	0.122	
		Vigorous physical activity	**0.071**	
		Total physical activity	**0.007**	
Have (2018)	Physical activity incorporated within mathematics lessons		Difference between control and intervention group	The physical activity intervention did not improve physical activity of body composition.
		BMI (kg/m^2^)	−0.24 (−0.8, 0.4)	
		Total physical activity (count/min)	−8.6 (−69.9, 52.7)	
		Cardiorespiratory fitness (m)	−12.3 (−46.9, 22.4)	
Ten Hoor (2018)	Strength exercise and monthly motivational sessions		Correlation between intervention and parameters as control intercept (95% CI)	After one year, the intervention group exhibited a greater reduction in fat mass compared to the control group. However, no notable differences were observed between the groups in terms of MVPA, sedentary behavior, or engagement in light physical activity.
		Sedentary	0.16 (−2.8–3.2)	
		Light physical activity	**0.03 (−2.2–2.2)**	
		MVPA	−0.14 (0.7–0.4)	
		Body fat %	**2.83 (0.1–5.6)**	
		Body Weight (Kg)	−0.36 (−1.5–0.8)	
Belton (2019)	Youth-Physical Activity Towards Health (Y-PATH)	MVPA	Effect of intervention on parameter **24.961 (18.005, 31.918)**	Y-PATH school-based intervention successfully increased MVPA in the intervention patients.
Jago (2019)	Action 3:30R		Difference in Means (95% CI)	No significant difference between the two arms in terms of MVPA and reduced sedentary time was observed.
		Weekday MVPA (mins)	−0.5 (−4.57, 3.57)	
		Overall mean MVPA (mins)	−0.75 (−4.49, 3.00)	
		Mean weekday sedentary (mins)	10.01 (−6.3, 26.31)	
Leahy (2019)	High-intensity interval training		Difference in Means (95% CI)	A statistically significant improvement in adiposity outcomes and physical fitness in older adolescents was observed.
		Cardiorespiratory fitness (laps)	**8.9 (1.7 to 16.2)**	
		Upper body muscular endurance (reps)	1.7 (−1.4 to 4.7)	
		Lower body muscular power (cm)	**10.1 (0.3 to 19.8)**	
		BMI (kg/m^2^)	**0.4 (0.1 to 0.6)**	
Müller (2019)	Multidimensional physical activity		Intervention effect; estimate b (95% CI)	The physical activity intervention showed significant improvement in adiposity measure; however, no significant impact on cardiorespiratory fitness was observed.
		Shuttle run (laps)	−0.56 (−4.67 to 3.56)	
		VO2max	−0.14 (−1.17 to 0.88)	
		BMI-z score	**−0.17 (−0.24 to −0.09)**	
		Skinfolds (mm)	**−1.06 (−1.83 to −0.29)**	
Pfeiffer (2019)	Girls on the Move		Linear mixed coefficient (95% CI) for change in parameters following intervention	There were no significant differences in BMI-z post-intervention. However, the intervention group exhibited a smaller increase in body fat percentage and a less pronounced decline in aerobic performance compared to the control group.
		BMI-z scores	**−0.02, (−0.05, 0.01)**	
		Body fat %	**−0.37, (−0.64, −0.10)**	
		VO2max	**0.20, (0.03, 0.36)**	
Robbins (2019)	Girls on the Move		Intervention effect compared to control (95% CI)	The intervention had no significant effect on increasing time spent by young girls on MVPA.
		MVPA	**–0.08 (–0.21, 0.05)**	
Seibert (2019)	CDC-based physical activity strategies		*p*-value between difference in both groups post intervention	The structured implementation of school-based CDC physical activity strategies did not result in greater improvements in cardiovascular fitness (CVF) compared to standard physical activity programs.
		PACER	0.05	
Seljebotn (2019)	Physically active lessons, homework and recess		Mean difference between groups [95% CI]	The intervention significantly improved the time spent in physical activity; however, it had no significant impact on adiposity.
		Sedentary (min/day)	**−13 [−26, 0]**	
		Light activity (min/day)	**−5 [−12, 3]**	
		MVPA (min/day)	**8** [3,13]	
		Steps per day	**940 [341, 1540]**	
		BMI	**0.1 [−0.4, 0.3]**	
Zhou (2019)	School physical education intervention, or after-school intervention, or both		Contrast coefficient between control and intervention (95% CI)	The physical activity intervention showed significant improvement in adiposity measure, cardiorespiratory fitness, and physical fitness.
		20 m shuttle run (laps)	**15.2 (12.3, 18.2)**	
		Broad jump	**17.0 (12.8, 21.3)**	
		50 m run (seconds)	**−0.4 (−0.6, −0.3)**	
		Sit-and-reach (cm)	**3.5 (2.5, 4.5)**	
		T test for agility (seconds)	**−1.0 (−1.2, −0.7)**	
		1 min sit-ups (counts)	**5.1 (3.3, 6.9); 0.16**	
		Plank support (seconds)	**31.8 (22.4, 41.2)**	
		Body fat (percent)	**−1.6 (−2.4, −0.8)**	
Breheny (2020)	The Daily Mile		Difference in adiposity between control and intervention group	The Daily Mile intervention caused no significant change in BMI z-scores and body fat % across the study population.
		BMI-z scores	**−0.036 (−0.085 to 0.013)**	
		Body fat %	0.56 (−2.15 to 3.27)	
Ketelhut (2020)	High-intensity interval training		Difference between control and intervention group	Students in the intervention arm have significant improvement in aerobic fitness (VO2max).
		AF (z-score)	**7.7 (2.3 to 13.2)**	
Maglie (2022)	Regular physical education and sports		*p*-value from baseline to post-intervention	Increased frequency and time for physical education and sports significantly improved body composition and physical activity levels in children.
		BMI percentile	**0.02**	
		Waist Circumference (cm)	**<0.0001**	
		Vertical jump (cm)	**<0.0001**	
		Standing broad jump (cm)	**<0.0001**	
		Rope jumps/min	**<0.0001**	
Marsigliante (2023)	Daily 10 min active breaks during lessons and recess		*p*-value from baseline to post-intervention	Addition of daily 10 min of physical activity significantly improved body composition and physical activity levels in children
		BMI percentile	**<0.001**	
		Waist Circumference (cm)	**<0.001**	
		Standing long jump (m)	**<0.0001**	
		Ruffier test	**<0.0001**	
		Sit-and-reach test	**<0.0001**	
Meng (2022)	Group A: High-intensity interval training Group B: Moderate-intensity continuous training	Change in parameter post-intervention		Both the physical activity interventions translated to significant improvement in body composition and cardiorespiratory fitness. High-intensity interval training was slightly superior to the moderate-intensity continuous training
		BMI	A: **22.7 ± 1.0** B: 23.2 ± 0.7	
		Body fat (%)	A: **36.2 ± 3.9** B: **34.4 ± 1.5**	
		Waist circumference (cm)	A: 78.8 ± 6.1 B: 78.5 ± 7.5	
		VO2max	A: **47.9 ± 2.6** B: **45.6 ± 2.1**	

AF: aerobic fitness; BMI: body mass index; CI: confidence interval; MVPA: moderate-to-vigorous physical activity; PE: physical education. Boldface indicates statistical significance with *p*-value < 0.05.

**Table 3:** Risk of bias for included studies.

Study	RS	AC	BP	BO	Attrition (Anth/Fit)	Attrition (PA/SB)	SR
Cohen (2015)	H	H	L	L	L	L	H
Jago (2015)	L	L	H	L	L	L	L
De Greeff (2016)	U	L	H	U	U	—	L
Hollis (2016)	L	L	H	U	H	H	L
Jarani (2016)	L	L	H	H	L	—	U
Lubans (2016)	L	L	H	U	L	U	U
Sutherland (2016)	L	L	H	L	L	L	L
Tarp (2016)	L	L	H	U	H	H	L
Donnelly (2017)	L	U	H	L	H	—	H
Lonsdale (2017)	L	L	L	L	—	H	L
Sutherland (2017)	L	L	L	L	—	U	L
Carlin (2018)	L	L	H	H	L	—	H
Have (2018)	L	L	H	L	L	L	L
Ten Hoor (2018)	L	L	H	H	—	H	H
Belton (2019)	H	H	H	H	H	H	H
Jago (2019)	L	L	H	H	L	L	L
Leahy (2019)	L	L	H	H	H	—	H
Müller (2019)	L	H	H	H	H	—	H
Pfeiffer (2019)	U	L	H	U	L	H	L
Robbins (2019)	L	L	L	L	L	L	L
Seibert (2019)	U	U	H	H	U	—	L
Seljebotn (2019)	L	U	H	H	L	L	H
Zhou (2019)	U	U	H	L	L	U	H
Breheny (2020)	L	L	H	L	L	—	L
Ketelhut (2020)	L	U	U	U	U	—	U
Maglie (2022)	H	U	H	U	L	L	L
Marsigliante (2023)	H	H	H	U	U	U	L
Meng (2022)	H	H	U	U	L	L	L

Legend: L = Low, H = High, U = Unclear, “—” = not applicable/not reported.

## List of Abbreviations

BMI	Body Mass Index
cpm	Counts Per Minute
HIIT	High-Intensity Interval Training
MVPA	Moderate-to-Vigorous Physical Activity
PACER	Progressive Aerobic Cardiovascular Endurance Run
RCT	Randomized Controlled Trial
SDGs	Sustainable Development Goals
VO2 max	Maximal Oxygen Uptake
WHO	World Health Organization
